# Unraveling water relations in growing fruit: insights from the epidermal growth regulation hypothesis

**DOI:** 10.3389/fpls.2024.1495916

**Published:** 2024-12-09

**Authors:** Norberto Gariglio, Carmina Reig, Manuel Agustí

**Affiliations:** ^1^ ICiAgro Litoral, UNL, CONICET, FCA, Esperanza, Santa Fe, Argentina; ^2^ Instituto Agroforestal Mediterráneo, Universitat Politècnica de València, Valencia, Spain

**Keywords:** fruit growth, physiological disorders, environmental factors, purple spot, fruit cracking, turgor pressure, osmotic potential, skin tissue

## Abstract

This review focuses on the intricate water relationships between internal and external tissues in growing fruits within the framework of the epidermal growth control hypothesis. It considers the components of water potential, including turgor pressure and osmotic potential of both internal and external tissues, taking into account factors such as fruit growth rate, sugar accumulation, cell wall metabolism, and climacteric. It also examines the effects of environmental conditions, genetic factors, and physiological influences in modifying water relations. By emphasizing the significance of skin tissue water potential components as indicators of growth stress, the review underlines their importance for a comprehensive understanding of water relations and associated physiological disorders in growing fruit.

## Introduction

Physiological disorders related to water dynamics represent a significant limitation to fruit growth, particularly fruit cracking, which limits both production and quality, especially in berries and drupes, but also in citrus (Matthews et al., 2009; Agustí et al., 2014; Winkler et al., 2015, 2016; Fischer et al., 2021), and purple spot, which can affect up to 60% of the fruit in loquat (Gariglio et al., 2002). There are several other physiological disorders related to fruit water relations, but they are beyond the scope of this manuscript.

The aim of this review is to examine the water relations between internal and external tissues in growing fruit through the lens of the epidermal growth control hypothesis. We examine current knowledge on the influence of environmental, genetic, and physiological factors on water relations, focusing on skin tissue as a key indicator of growth stress associated with physiological disorders.

Fruit development involves processes of cell division and expansion after fertilization. It is regulated by distinct transcriptional patterns and complex regulatory systems involving genetic, epigenetic, and hormonal controls at all developmental stages (Teyssier et al., 2015). Traditional assessment of fruit growth is based on dry matter accumulation and changes in cell enlargement. Both cell division and cell enlargement exhibit a sigmoidal or double sigmoidal growth pattern, depending on the species, that develops over time, typically in fleshy fruits (Rodriguez et al., 2019).

Fruit dry matter accumulation is the result of photoassimilate transport through the phloem by mass flow, a phenomenon elucidated by Mason and Maskell (1928). The pressure gradient resulting from the altered sugar concentration between source and sink tissues facilitates phloem sap transport, allowing sink organs to modulate mass flow rates through various metabolic enzymes, sugar transporters, and transcriptional and post-translational regulation (Sun et al., 2022; Ren et al., 2023). In summary, it is possible to increase crop yield by increasing source capacity and carbohydrate production in leaves or by increasing the use of photoassimilates in sink tissues (Driesen et al., 2023). In light of this, a number of agronomic practices have been developed and adopted by farmers to improve assimilate allocation to fruit, thereby increasing both the quantity and quality of their crops. These practices include pruning (Araújo-Ferreira et al., 2016), irrigation, fertilization, fruit thinning (Singh-Sidhu et al., 2022), modification of flowering intensity (Agustí et al., 2022), and the use of growth regulators (Gill et al., 2023), among others.

## Dynamic interaction between internal and external tissues in growing organs

A poorly explored facet of fruit growth is the relationship between internal and external tissues within a growing organ, mainly because of their different growth capacities. According to Kutschera and Niklas (2007), expansion of the internal tissues leads to elongation of the external tissues. Specifically, the inner tissues act as the primary force driving elongation, while the outer cell layers impose a mechanical constraint (Kraus, 1867). Consequently, this dynamic results in the generation of tension or stress within the outer cell layers due to the expansion of the internal tissues. The importance of the inner tissues in organ growth was highlighted by their active state of tension, which differed from that of the outer tissues. This led to a hypothesis that emphasized the role of the epidermis in growth regulation, the so-called “epidermal growth control hypothesis” (Kutschera and Niklas, 2007). This hypothesis focused the study on the architecture and properties of the epidermal cell walls, the structure and function of the cuticle, and the modification of these properties by hormonal actions, especially the changes that occur during fruit ripening (Kutschera and Niklas, 2007).

## Water potential components and their role in cell expansion

Parenchyma cells with a thin primary wall are the predominant cell type in fleshy fruits. In these cells, the turgor pressure (P) resulting from osmotic water uptake provides the necessary force to induce plastic deformation of the cell walls, allowing cell expansion and, consequently, the permanent increase in size or growth of different organs (Ortega, 2023). Plastic deformation occurs only when the turgor pressure exceeds a critical value (Pc), according to the following equations:


(1) $$\text{Relative volumetric plastic deformation rate of the cell wall }\left({\text{h}}^{-1}\right)\;=\phi \left(\text{P}–\text{Pc}\right)$$

where *ϕ* = coefficient of irreversible extensibility of the cell wall (h^−1^ MPa^−1^), P = turgor pressure (MPa), and Pc = critical turgor pressure for plastic extension (MPa).

Consequently, the water potential (Ψ_a_) of the parenchyma cells in fleshy fruits is controlled by two primary components. Firstly, the tissue pressure (P) within the fruit, due to the cell wall and the skin of the fruit, limits the expansion of both the cell and the flesh. Secondly, the osmotic potential (π) induced by the presence of soluble metabolites; hence, Ψ_a_ = P – π. As a result, the osmotic potential favors the influx of water into the cell, whereas the pressure potential exerts a restrictive influence. While elevated metabolite concentrations are uncommon in plant vegetative organs, they are a common phenomenon during the ripening of fleshy fruits (Jia et al., 2020).

## Fruit ripening: growth rate, water potential, osmotic adjustment, and cell wall dynamics

According to the epidermal growth control hypothesis, the regulation of fruit growth is controlled by the epidermal tissue (Kutschera and Niklas, 2007) according to Equation 1. The cell wall strength and cell–cell adhesion of the fruit epidermis are not constant, but change over time (Canton et al., 2020; Shi et al., 2023). Therefore, the critical turgor for fruit growth and the coefficient of irreversible extensibility of the cell wall are subject to modification throughout the course of fruit development (Ortega, 2023), resulting in a corresponding change in fruit growth rate. However, the period of most pronounced change in cell wall structure, and consequently on fruit growth rate, is during fruit ripening. This involves a variety of physiological, structural, and metabolic changes that ultimately lead to the development of edible fruit. The regulation of this process occurs at different molecular levels, with phytohormones, transcription factors, and epigenetic modifications all playing a crucial role (Perotti et al., 2023).

Ethylene has been highlighted as a key phytohormone in the ripening of climacteric fruits, while abscisic acid also plays a central role in non-climacteric fruits (Li et al., 2021; Li et al., 2022). During the ripening process of climacteric fruits, changes in primary cell wall metabolism are mainly associated with the induction of several gene families, including expansins (EXPs), xyloglucan endotransglucosylase (XET)/hydrolases (XTH), and endo-1,4-β-glucanases (EGase or Cel) (Shi et al., 2023). While some changes in cell wall composition and structure are common to different species, others are species-specific (Canton et al., 2020). Interestingly, auxin and other agents can modulate cell wall growth rates in living plants very rapidly, within minutes or even seconds. Furthermore, this rapid response could be controlled by altering wall pH, which would activate or inactivate expansins, the proteins responsible for acid-induced wall extension in growing tissues (Cosgrove, 2000). In addition, cellulose can achieve a much higher degree of structural order, or crystallinity, which has a strong effect on the mechanical properties of cell walls, as the ratio of crystalline to amorphous cellulose is associated with the rate of extension (Bringmann et al., 2012).

During ripening, the metabolism of climacteric fruit also induces the degradation of polymers such as starch and cell wall polysaccharides, leading to changes in cellular water status due to catabolism. As a result, the cellular concentrations of metabolites increases, resulting in a lower cellular osmotic potential, which increases water uptake and turgor pressure, allowing the fruit to maintain a high growth rate even under adverse conditions (Blum, 2017; Miranda Fernandes et al., 2018). The contribution of metabolites to osmotic adjustment also includes acids, phenolics, amino acids, soluble pectins, and minerals, all measured as part of the soluble solids content (Sharma et al., 2019; Hou et al., 2020).

However, sugar metabolism and accumulation during ripening are also important in non-climacteric fruits, as observed in loquat, where almost 90% of the total sugar accumulation takes place within a 15-day period from color break (Gariglio et al., 2003a), and this period coincides with the phase of higher fruit growth rate (Gariglio et al., 2003b). Similar trends were observed in grapes (Matthews et al., 2009) and sweet cherries (Schumann et al., 2014). 

As observed in fruits, the enzymatic modification of the cell wall of the epidermal tissue is also responsible for modifying of the growth rate of vegetative tissues, such as the leaves of *Lolium temulentum* (Bacon et al., 1997). In *Cucurbita pepo*, the periods of maximum growth rate in both fruit and leaves coincide with the highest levels of ascorbic acid oxidase (AAO) in the epidermis (Lin and Varner, 1991). This enzyme softens the cell walls, thereby facilitating an increase in growth rate. Furthermore, the epidermal growth control hypothesis has been proposed to explain the growth tension between internal and external tissues of the hypocotyl of sunflower seedlings (Kutschera and Niklas, 2007).

In summary, specific phytohormones and enzymes play a crucial role in modulating the water relations and, consequently, the growth rate of growing tissues by altering the structure of cell walls. These modifications impact pivotal parameters of the water relations (see Equation 1), including the coefficient of irreversible extensibility of the cell wall and the critical turgor pressure required for plastic expansion. Furthermore, they contribute to the catabolism of reserve substances, thereby triggering osmotic adjustment processes. By influencing these factors, phytohormones and enzymes contribute to the dynamic regulation of growth processes in response to environmental and developmental cues.

## Water relationships between internal and external tissues during fruit growth

The epidermal growth control hypothesis establishes the occurrence of stress between internal and external tissues during growth; however, the water potentials and their components in both internal and external tissues have not been thoroughly studied. Nevertheless, some studies have indirectly investigated the evolution of water potentials in flesh tissues, and their analysis is crucial for understanding this physiological process in fruit. Rain-induced cracking of sweet cherry fruit significantly limits its global production and has been the subject of extensive research (Winkler et al., 2016). An accepted conceptual framework to explain this phenomenon is the critical turgor model, originally proposed by Considine and Kriedemann (1972), with a specific focus on grape (*Vitis vinifera* L.) berries. According to this theoretical model, the flesh of the berry is compressed by an elastically stretched skin. As the fruit absorbs water, the internal pressure within the fruit increases. Once the critical threshold is reached, the fruit skin is overstressed beyond its elastic limit, resulting in cracking (Considine and Kriedemann, 1972).

However, a study in which fruits of 19 sweet cherry cultivars were incubated in water to analyze water uptake and cracking contradicted the notion that cracking is a simple function of the amount of water absorbed, as proposed by the critical turgor model (Winkler et al., 2016). In the following years, extensive research was carried out to improve the understanding of water relations in cherries in order to explain the cracking process (Schumann et al., 2014). Tissue water potential and fruit turgor showed a decrease 55 days after full bloom and remained low until maturity. In contrast to the critical turgor model, cell turgor decreased from the beginning of stage III of sweet cherry fruit growth, dropping from 350 to 25 kPa within the same period. Consequently, in the absence of cell turgor, both water potential and osmotic potential tended to converge to similar values. Surprisingly, fruit growth continued at high rates despite the significant reduction in cell turgor (Schumann et al., 2014). Based on these results, the hypothesis that cracking is induced by an increase in tissue turgor was rejected (Schumann et al., 2014). In another study of 17 European plum cultivars, cell turgor pressure decreased significantly from 0.33 to 0.35 MPa 78 days after full bloom to 0.02 MPa at harvest. This final measurement was remarkably low compared with the markedly negative osmotic potential (<−3.00 MPa) and water potential recorded at maturity, both of which reached similar values at this time. Furthermore, cell turgor in European plum was found to be independent of osmotic potential (Knoche and Grimm, 2022). These results showed a parallel trend to those mentioned for sweet cherry (Schumann et al., 2014).

In Cabernet Sauvignon berries grown in a greenhouse, cell turgor declined significantly, from a peak of 0.30 MPa at 48 days after anthesis to approximately 0.05 MPa at 60 days after anthesis, and this level was maintained throughout ripening (Matthews et al., 2009). During the initial phase of turgor decline, from 0.30 to 0.16 MPa, there was only a marginal increase in soluble solids, but cell turgor continued to decline, increasing to 3.6°Brix within 3 days. This trend continued, indicating that significant sugar accumulation did not begin until cell turgor reached about 0.10 MPa or less. In the case of Pinot Noir, the pattern of cell turgor is similar to that described for Cabernet Sauvignon (Matthews et al., 2009).

In loquat fruit, the flesh turgor is consistently low, remaining below 0.15 MPa throughout the growth period. Interestingly, in a similar context, turgor becomes almost non-existent around the time of color break, a phase when the fruit reaches its highest growth rate (Gariglio et al., 2008a; Reig et al., 2016). In contrast to observations in sweet cherry (Schumann et al., 2014), European plum (Knoche and Grimm, 2022), and grape (Matthews et al., 2009), there is no significant decrease in turgor, osmotic potential, and water potential in loquat at the beginning of phase III of fruit growth (Gariglio et al., 2008a).

## Water status of the skin tissue

Using a miniaturized pressure probe, Kutschera and Niklas (2007) observed that cell turgor in the outer tissues of sunflower hypocotyls of 4-day-old dark-grown seedlings is comparable to that of the inner tissues (0.48–0.49 MPa). However, there is a marked difference in osmotic potential between the epidermal layer (0.63 MPa) and the internal tissues (0.56 MPa). Consequently, the water potential is more negative in the outer tissues (−0.14 MPa) compared to the inner tissues (−0.08 MPa), meaning that the water potential gradient drives the movement of water from the inner tissues into the epidermis (Kutschera and Niklas, 2007).

A limited number of studies have been identified that specifically investigate water potentials and their components within the internal and external tissues of growing fruits. One such investigation, which sheds light on the physiological origins of loquat purple spot, is reported in the work of Gariglio et al. (2008a). This study focuses on the analysis of water potential, providing an important perspective for understanding the underlying physiology associated with water relations between internal and external tissues.

Purple spot appears as an extensive area with a slightly depressed surface, characterized by a purple color and irregular shape, affecting only the epidermal tissue of the fruit. The affected areas begin in the deepest layers of the skin cells and progress to the more superficial cell layers. The cuticle shows no signs of damage and its water permeability is unaffected. Therefore, water loss from the fruit to the atmosphere cannot be considered as the cause of the skin dehydration that characterizes purple spot (Gariglio et al., 2002). Subsequent research has shown that epidermal dehydration results from a change in the water balance between the flesh and the rind, which occurs at the same time as fruit color breakdown. This period is characterized by both a significant increase in sugar accumulation and a high fruit growth rate. The dehydration process is also influenced by cultural practices, such as the intensity of fruit thinning, and environmental factors, such as low temperature and exposure to sunlight. These factors affect the assimilation and partitioning of sugars and minerals, favoring the flesh and increasing the solute concentration gradient between the two tissues (Gariglio et al., 2008b). In conclusion, the purple spot of the loquat fruit is a physiological disorder explained by the tension between internal and external tissues, as postulated by the epidermal growth control hypothesis. Therefore, their results have the potential to shed light on this hypothesis and the water relations established in this context.

**Figure 1:**
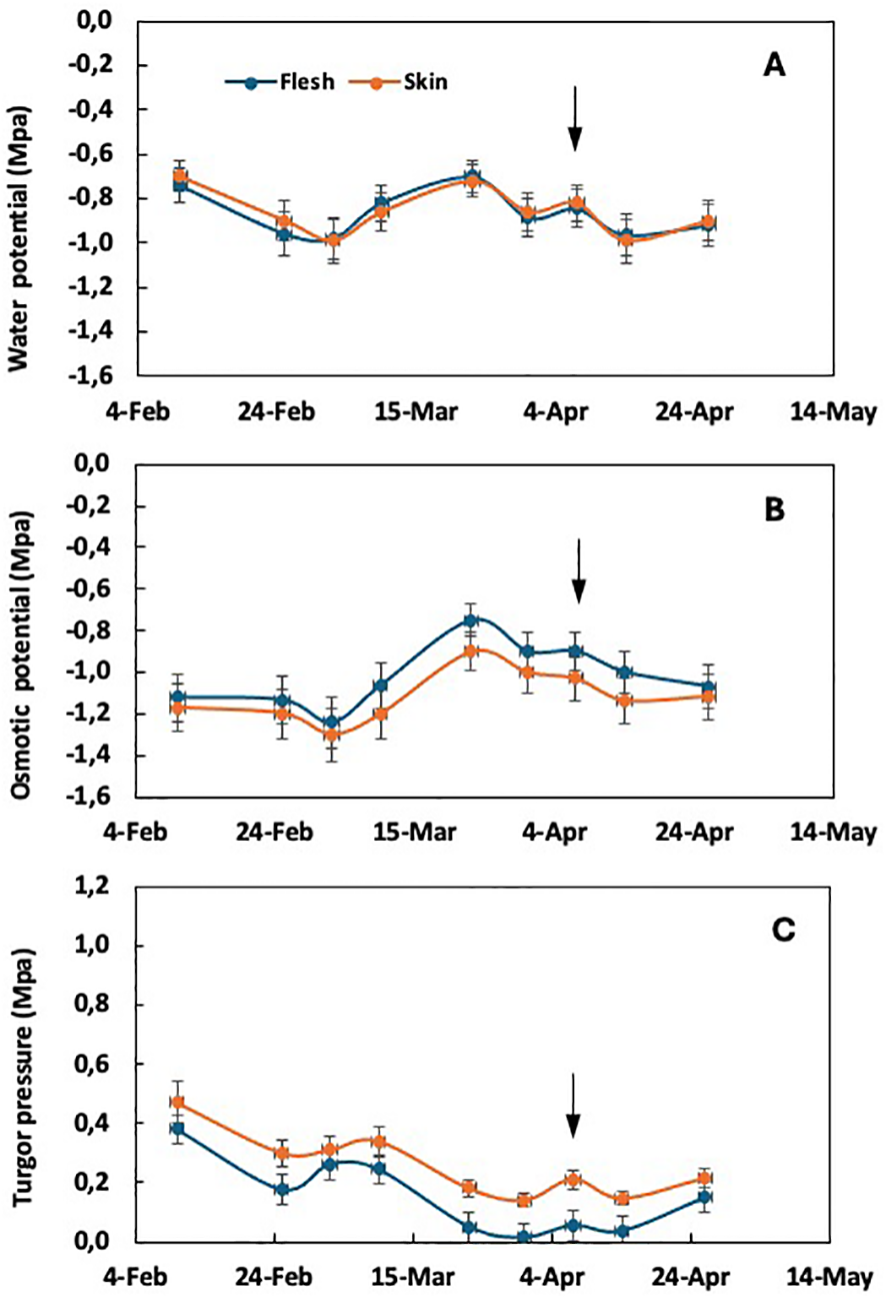
Time course of water potential **(A)**, osmotic potential **(B)**, and cell turgor pressure **(C)** in the flesh and skin tissue of loquat through fruit growth in non-fruit-thinned plants. Arrows indicate the time of color break. Adapted from Gariglio et al. (2008a).

**Figure 2:**
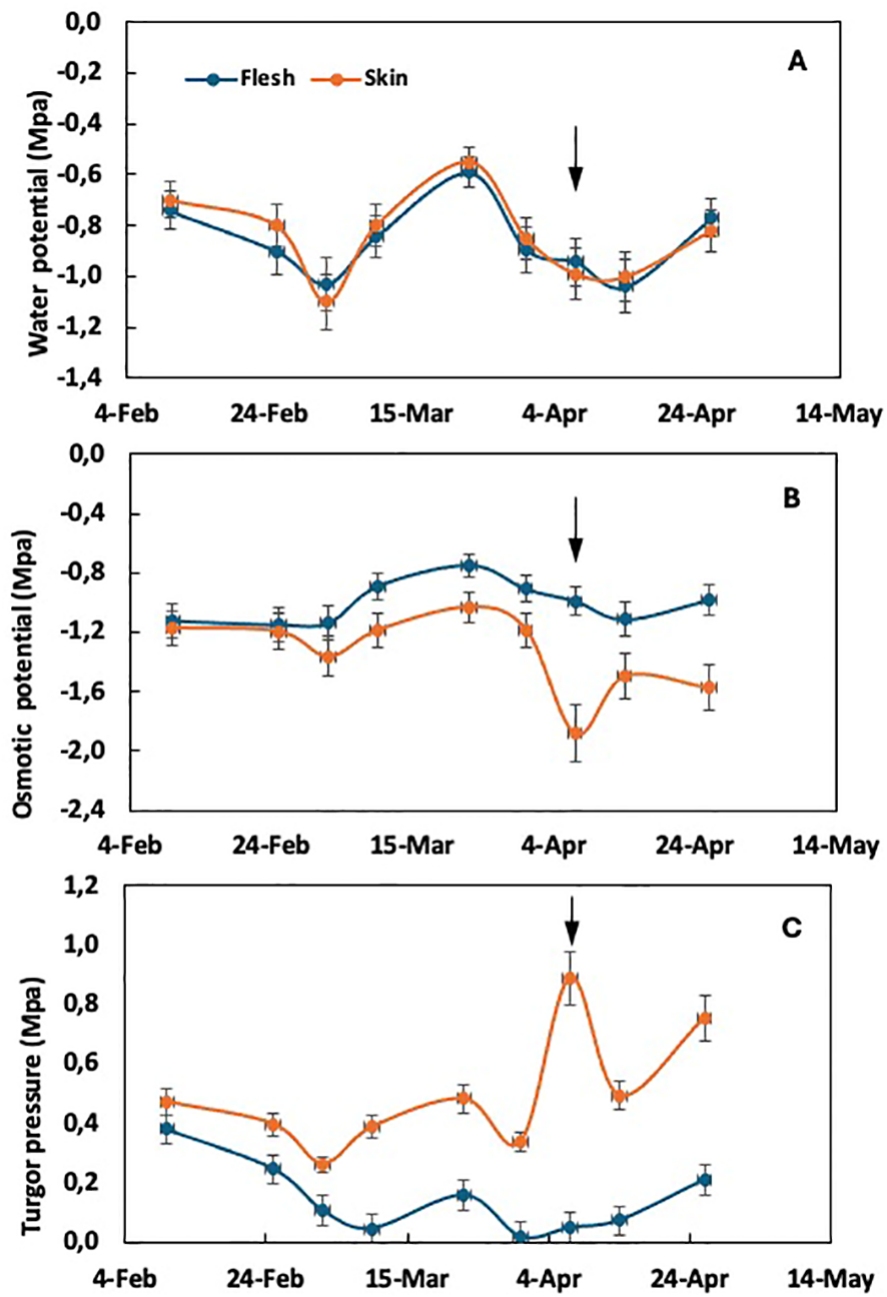
Time course of water potential **(A)**, osmotic potential **(B)**, and cell turgor pressure **(C)** in the flesh and skin tissue of loquat through fruit growth in plants thinned to one fruit per panicle. Arrows indicate the time of color break. Adapted from Gariglio et al. (2008a).

As the incidence of purple spot increases with the intensity of fruit thinning, a comparative analysis was carried out between plants thinned at different rates of fruit per panicle. In particular, unthinned trees showed no incidence of purple spot, whereas plants thinned at one fruit per panicle showed a significant fruit damage rate of 34%. Intermediate incidence levels were observed in plants thinned at five and three fruits per panicle (Gariglio et al., 2003a). Comparing the extreme treatments of fruit thinning, the average water potential of both flesh and skin tissues did not show significant differences, but varied with the sampling date. This is true for both unthinned plants (**Figure 1A**) and plants thinned to one fruit per panicle (**Figure 2A**). In the absence of purple spot risk (unthinned plants), the osmotic potential of the skin tends to be slightly lower than that of the flesh tissue (**Figure 1B**), while the turgor remains slightly higher throughout the fruit growth period.

In plants thinned to one fruit per panicle, where the susceptibility to purple spot is maximal, the evolution of water potential in both flesh and skin tissues does not show differences (**Figure 2A**), as observed in unthinned plants. However, significant changes are observed in the components, osmotic potential and turgor pressure. The osmotic potential of the skin tends to decrease compared to the flesh tissue from the end of February, coinciding with the appearance of purple spot symptoms. At this time, a sudden drop in skin osmotic potential is observed (**Figure 2B**), corresponding to an abrupt increase in turgor pressure (**Figure 2C**) within the skin tissue. The drop in osmotic potential observed at color break in fruit from thinned trees (**Figure 2B**) suggests that either water uptake lags behind sugar accumulation in the skin or there is a loss of water from the skin to the flesh in fruit from thinned trees. This dehydration is a characteristic symptom of purple spot (Gariglio et al., 2002).

Significantly, the stress associated with growth according to the epidermal growth control hypothesis is evident in the solute potential and turgor pressure of the skin tissue rather than in the flesh. This distinction is noteworthy as most studies of fruit growth tend to focus on the water potential and its component in the flesh tissue.

## Xylem reflux

Similar to the modulation observed in other fleshy fruits, the primary water import pathway of grape (*Vitis* sp.) berries undergoes a transition from xylem to phloem at the onset of veraison. Consequently, berry water uptake through the xylem is reduced, although the structural integrity of the berry xylem remains unaffected and unhindered throughout the ripening process (Zhang and Keller, 2016).

**Figure 3:**
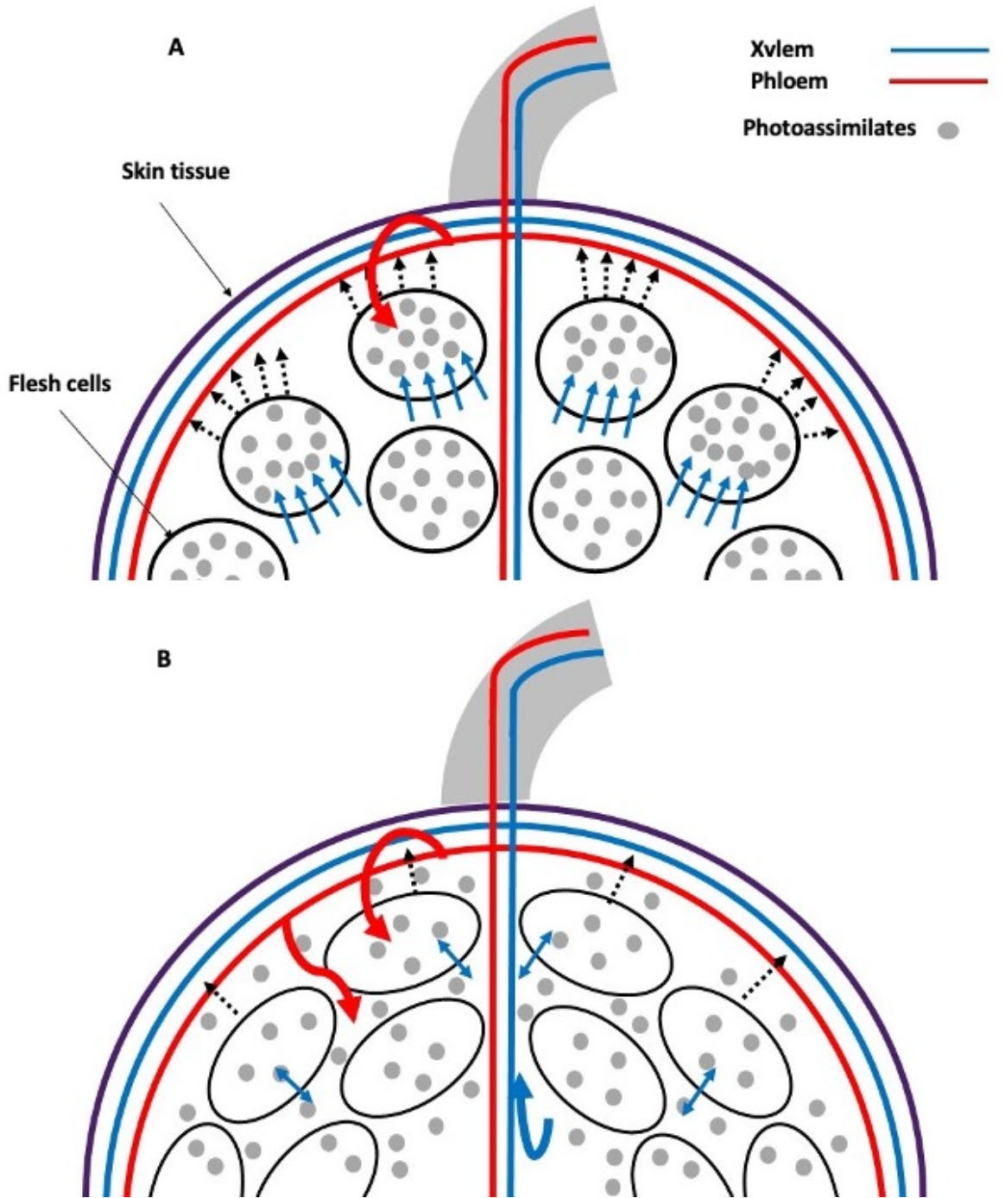
Xylem backflow in grape: phloem photoassimilates in sink tissues are unloaded preferentially symplasmically **(A)**. However, during ripening, this process shifts to apoplastic pathways **(B)**. The unloading of sucrose symplastically results in the accumulation of this substance within flesh cells, which, in turn, causes osmotic water uptake and an increase in turgor pressure. Conversely, when sucrose is partially unloaded via apoplastic pathways, it accumulates in the intercellular spaces of the flesh tissue. Consequently, when there is an excess influx of phloem water and sugars into the fruit, they are recirculated through the xylem (xylem backflow) as turgor pressure slightly exceeds the resistance offered by the external tissue. Light blue arrow: cellular osmotic water uptake; dashed arrow: turgor pressure; curved arrow: excess photoassimilates and water phloem recirculated through the xylem (xylem backflow). Created based on the results and discussion of Zhang and Keller (2016).

Sucrose is unloaded from the phloem in sink tissues either apoplasmically or symplasmically (Ruan, 2014). Apparently, there is a change in the primary water transport pathway in grape berries with a transition from symplastic to apoplastic phloem unloading at the onset of ripening (Zhang et al., 2006). This hypothesis was confirmed by studying the movement of xylem mobile dyes in grape berries and root pressure treatments. Modeling studies have shown that some phloem-derived water contributes to both berry growth and transpiration, with the excess being recirculated through the xylem (xylem reflux) (**Figure 3**). Restricting the release of water through the xylem and/or the skin limits the accumulation of solutes in the berry and its color change. This mechanism can be viewed as a strategy to increase the berry sink strength and promotes normal grape ripening and may be particularly important during periods of rapid sugar accumulation and under environmental conditions that limit berry transpiration (Zhang and Keller, 2016).

## Hypothesis on the mechanisms of skin tissue dehydration leading to purple spot in loquat fruits

At the color break stage, loquat fruits exhibit their highest growth rate, which is 2.5 times faster in fruits from plants thinned at one fruit per panicle compared to unthinned plants (Gariglio et al., 2003a). The concentration of total soluble carbohydrates in flesh tissue (over dry mass) at color break was also 3.52 times higher in plants thinned at one fruit per panicle compared to unthinned plants (Gariglio et al., 2003a). In peel tissue, the concentration of total soluble carbohydrates at color break is less than a quarter of that in flesh tissue. In grapes, the ratio of sugar concentration between flesh and skin tissue is in the range of 2 to 3 during fruit development (Zhu et al., 2017).

**Figure 4:**
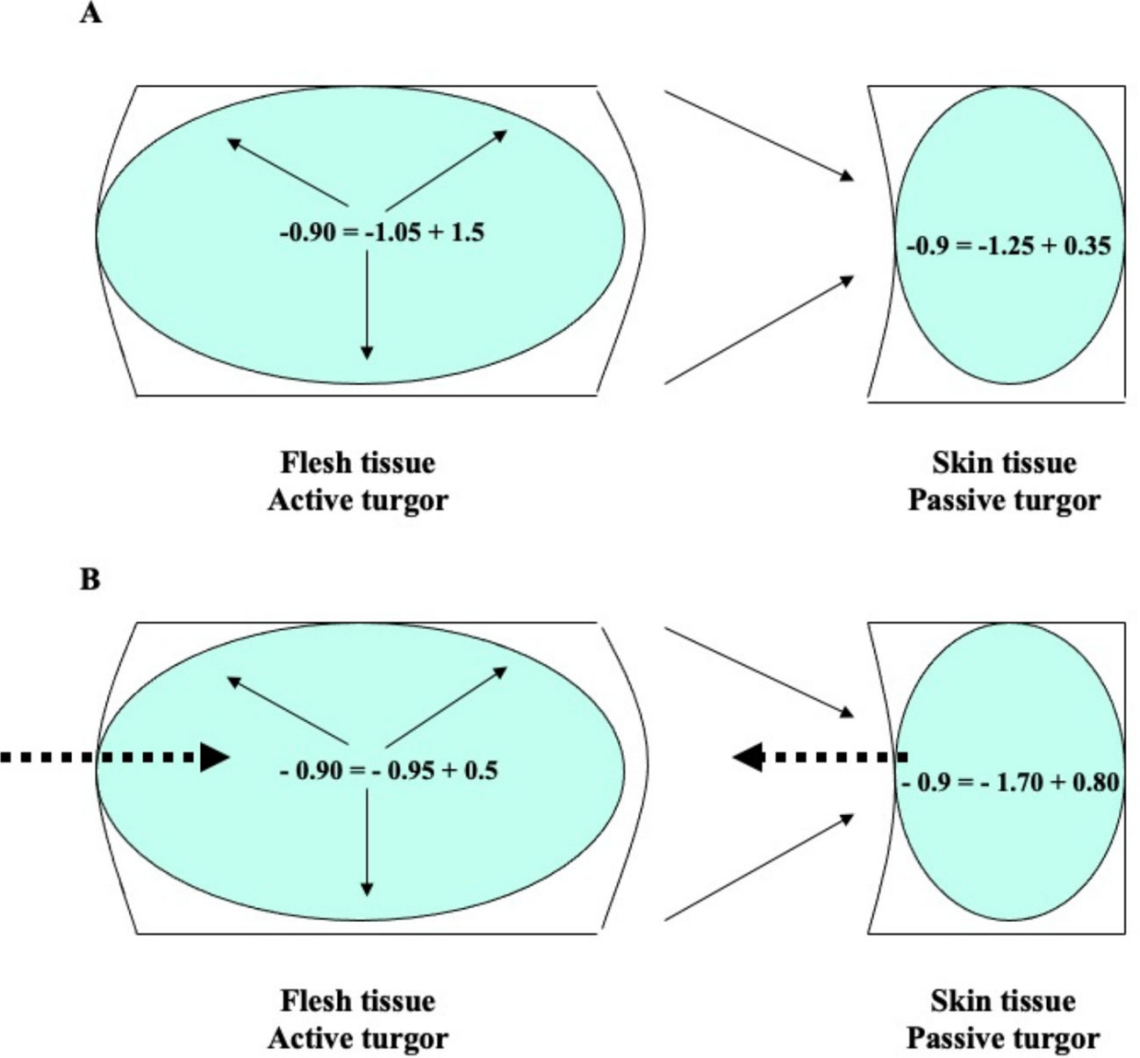
Water potential (Ψ_a_), osmotic potential (π), and cell turgor pressure (P) in flesh and skin tissue of loquat fruit at color break with low **(A)** and high **(B)** risk of purple spot. Ψ_a_ = π + P. Dashed arrows indicate water flow; solid arrows indicate the origin of turgor. In the flesh, turgor is a consequence of osmotic water uptake (active turgor); in the skin, turgor is a consequence of flesh expansion reducing skin cell volume (passive turgor).

In this context, the progressive weakening of the peel tissue observed in loquat fruits (**Figures 1**, **2**) can be attributed to the complex biochemical changes associated with the onset of ripening. This phenomenon facilitates the influx of water with minimal restriction, given the low turgor and relatively constant osmotic potential of the flesh tissue at color break (**Figure 4**), despite the increased sugar accumulation (expressed as dry weight). This phenomenon explains the exponential growth of loquat fruit from the onset of color break to harvest.

As a result of the expansion of the flesh, the cell volume of the external tissues is compressed, leading to an increase in their turgor and, consequently, their water potential (**Figure 4**). The equilibrium is restored by the migration of water from the outer tissues (higher water potential) to the inner tissues (lower water potential). As a result, there is a gradual decrease in osmotic potential (due to water loss) and a simultaneous increase in turgor in the skin of loquat fruits. In some cases, the intensity and duration of this growth stress can lead to irreversible cell dehydration and collapse of the skin cells, resulting in symptoms of purple spot (**Figure 4B**). The extreme values of the osmotic potential recorded in the fruit skin during pre-down measurements are −2.5 MPa (Gariglio, 2001; Gariglio et al., 2008a).

In low crop load conditions, the osmotic potential of the peel tissue is dependent on the rate of sugar accumulation, the sugar concentration gradient (dry basis) between the flesh and the peel, and the intensity of the fruit growth rate. The combined effect of these factors determines the risk of irreversible peel dehydration (Gariglio et al., 2008a, b). Conversely, in situations of high crop load, there is no correlation between the peel osmotic potential and any of the aforementioned factors. This indicates that the intensity of these factors is insufficient to influence the peel’s hydration status (Gariglio, 2001; Gariglio et al., 2008a). In contrast to the previous result, a more negative osmotic potential of the flesh compared to the skin tissue was observed in sweet cherry (Grimm and Knoche, 2015). In the case of tomatoes, the osmotic potential of the flesh was found to be slightly lower than that of the skin tissue. Additionally, a low cell turgor (0.04-0.08 MPa) was observed in the skin (Ikeda et al., 1999; van Ieperen et al., 2005).

## Physiological significance of turgor pressure in internal vs. external tissues of growing fruit

The physiological significance of cell turgor pressure differs between flesh and skin tissue. In flesh, turgor pressure is generated by osmotic water uptake (**Figure 4**), which is a consequence of sugar accumulation (Ortega, 2023). Additionally, since the internal tissue is limited in its expansion, it is in a state of active tension in accordance with the growth control hypothesis (Vines, 1886; Kutschera and Niklas, 2007).

In the outer tissues, the pressure exerted by the expanding flesh tends to reduce the skin cell volume (**Figure 4**), which indirectly increases turgor. Consequently, the skin tissue is in a state of passive tension (Vines, 1886; Kutschera and Niklas, 2007). In light of these concepts, it can be argued that in tissues that are in a state of active tension, the flesh, the turgor pressure should be referred to as “active turgor pressure” or “true turgor pressure”. Conversely, in external tissues, turgor results from a decrease in cell volume due to the driving forces of the internally expanding tissue. In this case, turgor should be referred to as “passive turgor pressure” or “false turgor pressure” (**Figure 4**). In the case of loquat fruit, passive turgor pressure is responsible for maintaining a high water potential of the skin cells even as the tissue dehydrates under conditions predisposing to purple spot (Gariglio et al., 2008a).

In sweet cherry, an osmotic potential gradient of 1.1 MPa was observed between the fruit skin and its flesh, with the osmotic potential being more negative in the flesh (Grimm and Knoche, 2015). The low turgor pressure measured in the flesh (Schumann et al., 2014) establishes a significant water potential gradient from skin to flesh (Grimm and Knoche, 2015). However, the process by which water potential equilibrium is achieved between the two tissues remains unclear. There is currently a lack of data available on apoplastic phloem unloading (Zhang et al., 2006) and the xylem reflux mechanisms (Zhang and Keller, 2016) in different species. It is therefore possible that a significant proportion of the solutes measured in the extracted juice from sweet cherry pulp (Grimm and Knoche, 2015) are apoplastic and therefore may not accurately represent the osmotic potential of the pulp tissue. In tomato, the study by Ikeda et al. (1999) showed no water potential gradient between flesh and skin tissue during the pre-dawn period. Nevertheless, an osmotic gradient was observed between the apoplastic and symplastic spaces, which could facilitate substantial water import into the cells. In practice, however, this import did not occur. This may be attributed to the presence of matrix potentials in the apoplastic space, resulting from capillary forces within the cell walls (van Ieperen et al., 2005).

The manuscript by Gariglio et al. (2008a) presents an original hypothesis that sheds light on the changes in water relations between the flesh and skin tissues of developing loquat fruits, and the prospective emergence of physiological disorders. In the present manuscript, the explanation and definition of the presence of passive turgor in the peel tissue is also and original physiological concept. The hypothesis and the introduction of the concept of passive turgor are of great importance as they provide a novel contribution to the understanding of the regulation of water dynamics in fruit under different crop load conditions.

## Factors influencing water relationships between internal and external tissues

### Fruit growth rate

According to the epidermal growth control hypothesis, stress between internal and external tissues occurs during organ growth. Under conditions of negligible or low growth rate, changes in water potential between internal and external tissues are not observed (see **Figure 1**). The appearance of purple spot is only observed under conditions characterized by a high fruit growth rate around the time of color break (Gariglio et al., 2002, 2008a, b).

Fruit cracking is a common physiological disorder associated with stress between internal and external tissues of growing fruits. The complexity of cracking is further compounded by the influence of a highly variable external environment, which makes it challenging to study even under controlled conditions (Santos et al., 2023). The probability of this phenomenon increases significantly under conditions of high fruit growth rate and around the time of fruit ripening, particularly in conjunction with rainfall (Mesejo et al., 2016; Wang et al., 2021; Micheloud et al., 2023). For example, cracking in tomatoes is most prevalent in the early morning or late afternoon, when growth rates and sap flow are at their highest (Guichard et al., 2001).

### Sugar and mineral accumulation and gradient

Sugar accumulation facilitates osmotic water uptake and provides the necessary force to induce permanent increases in size or growth of various organs (Ortega, 2023). Cultural practices such as fruit thinning allow the manipulation of source–sink relationships, thereby enhancing the partitioning of dry matter to fruit and maximizing commercial yield (Costa et al., 2018; Falchi et al., 2020).

Vascular bundles, both xylem and phloem, are responsible for delivering essential nutrients and water to either the flesh or seed of the fruit. The main bundles give rise to numerous branches that diverge and anastomose extensively throughout the flesh tissue (Falchi et al., 2020; Hou et al., 2021). This results in an accumulation of sugars and minerals in the inner tissues, which creates an imbalance with the outer tissues. The unequal distribution of minerals and assimilates between the flesh and peel, which occurs under conditions of high fruit growth rate, is responsible for the water imbalance that leads to the appearance of purple spot in loquat fruit (Gariglio and Agustí, 2005; Gariglio et al., 2008b). It is noteworthy that the foliar application of various salts, including calcium nitrate, calcium chloride, Ca-EDTA, ammonium nitrate, and potassium nitrate, conducted 2 weeks prior to the color break, has exhibited the capacity to replenish the osmotic strength of the skin tissue. These treatments have demonstrated a significant reduction in the proportion of fruit affected by purple spot (Gariglio et al., 2005).

In the case of cracking, it has been observed that the resistance of jujube varieties to cracking was found to be related to the content of reducing sugars, total soluble sugars, and cellulose, but not to the starch content. Crack-resistant varieties were found to have lower levels of total soluble sugars than crack-sensitive varieties (Li et al., 2020). Instead, it has been proposed that cracking is caused by the accelerated and uncoordinated growth and expansion of internal tissues, that exceed the mechanical resistance of the fruit skin (Mesejo et al., 2016; Butani et al., 2019; Wang et al., 2021). Therefore, it is essential that any study or interpretation of this physiological disorder takes into account the different levels of sugar accumulation and fruit growth rates.

The phloem unloading mechanisms of sucrose and the apoplastic pathway have been documented (Ruan, 2014; Zhang and Keller, 2016). However, xylem backflow in fruit flesh tissue, which is a consequence of apoplastic phloem unloading, has only been demonstrated in a limited number of species. As a result, our understanding of this physiological process remains incomplete, and its role in fruit growth, ripening, and the prevention of physiological disorders remains unclear. However, there is evidence that the occurrence of symplastic and apoplastic mechanisms depends on developmental stage and genotype (Falchi et al., 2020). Despite its significant impact on source–sink relationships, sink strength, and sugar accumulation (Zhang and Keller, 2016), it is essential to investigate this process in each fruit species to interpret its species-specific role in water relations between internal and external tissues of growing fruits. According to Schumann et al. (2014), apoplastic solute accumulation in the fruit, the physiological mechanism that allows xylem backflow, prevents a catastrophic increase in cell pressure and subsequent fruit cracking in grapes. In peach flesh, xylem sap, together with solutes and water unloaded from the phloem into the apoplast, is transported predominantly through the flesh by the bulk flow of water (Morandi et al., 2007, 2010). However, what happens when the conditions that maintain backflow change significantly? For instance, this may occur during rainfall or when the fruit is detached from the tree. What changes occur in the solute and water balance under these conditions, and how do they affect the occurrence of cracking or the development of purple spot?

It is important to note the distinction between fruit cracking and purple spot, particularly in loquat and sweet cherry. It is worth noting that purple spot on loquat fruit does not occur when the fruit is removed from the tree. Rather, it is specific to certain stages of fruit growth, such as color break (Gariglio et al., 2002). In contrast, sweet cherry cracking is primarily observed under rainy conditions during the ripening and harvesting season, or when detached fruits are incubated in water solutions (Winkler et al., 2015; Knoche and Winkler, 2019).

The authors put forward the “zipper hypotheses” to explain cherry cracking. These hypotheses posit that stress between internal and external fruit tissues during phase III of fruit growth, but particularly in the cuticle, is a key factor. This is due to negative regulation of genes involved in the synthesis of cutin and waxes. Skin stress causes microcracks in the cuticle, which, when combined with moisture, leads to a further intensification of these microcracks and a concentration of water absorption in a specific region of the fruit surface. The water moves towards the flesh cells due to their lower water potential and thin cell walls, causing them to rupture and release cellular contents into the apoplast at concentrations comparable to those in the symplast. Consequently, cell turgor is either reduced or lost. Malic acid has been shown to effectively remove calcium from cell walls, thereby reducing their strength and increasing the permeability of plasma membranes, causing a loss of adhesion between adjacent cells. These results in swelling of the cell walls, particularly the pectins of the middle lamella, separation of adjacent cells along the cell wall, and ultimately skin rupture, causing the fissure itself to widen (Knoche and Winkler, 2019).

### Mechanical resistance of the fruit skin

As discussed in the previous section, fruit growth rate is closely related to flesh sugar concentration. However, in the case of loquat fruits, variations in pulp sugar concentration can explain variations in growth rate when comparing fruits at the same stage of development. It is important to emphasize that flesh sugar concentration does not consistently explain the changes observed in the growth rate during fruit development, particularly the sudden increase measured at the onset of color break (Gariglio et al., 2008b). The cell wall metabolism of the peel tissue, as discussed previously, provides a more comprehensive explanation for the variation in growth rates during fruit development. This is because the observed metabolic changes contribute to the variability of both cell wall strength and intercellular adhesion in the fruit epidermis, rather than remaining constant over time (Canton et al., 2020; Shi et al., 2023).

The balance resulting from factors such as fruit growth rate, skin strength, and sugar accumulation is likely to influence the water potential and its components between internal and external tissues, potentially affecting susceptibility to physiological disorders (Gariglio et al., 2008a). For example, in sweet cherry (Schumann et al., 2014; Grimm and Knoche, 2015), European plum (Knoche and Grimm, 2022), and grape (Matthews et al., 2009), both the water potential and the osmotic potential of the flesh tissue decrease during fruit ripening. In contrast, in loquat (Gariglio et al., 2008a), the stability of both osmotic and water potentials is maintained. This stability indicates a delicate balance between sugar accumulation and water influx in loquat, with a complete dilution of sugars (Gariglio et al., 2008a). On the other hand, the decrease in osmotic potential in sweet cherry, European plum, and grape indicates a restriction or delay in water uptake, coupled with a higher sugar accumulation than water influx, leading to an increase in total soluble solids content, as exemplified by grape with a rapid increase of 3.6°Brix in only 3 days (Matthews et al., 2009). However, and in the absence of turgor in the flesh, it becomes difficult to explain the water balance between the flesh and the skin tissue. Grimm and Knoche (2015) suggested that this water potential and osmotic gradient is a driving force for the diffusion of osmolytes from flesh to skin, and for the flow of water in the opposite direction from skin to flesh. However, this water movement apparently did not appear to occur, as the osmotic potential of the skin tissue did not decrease.

The evolution of the water potential components in the skin tissue has yet to be extensively studied. In loquat, it has been demonstrated that growth-related stress is markedly more pronounced in the skin than in the flesh tissue (**Figures 2**, **4**), with a reduction in osmotic potential observed in the former but not in the latter. In contrast, sweet cherry shows a more negative solute potential in the flesh in comparison to the skin (Grimm and Knoche, 2015). The discrepancy between these species may be attributed to the strength of the skin in each case, which induces a different water balance between internal and external tissues. Consequently, skin strength could potentially represent an additional variable influencing this water balance in this context.

### Climacteric and non-climacteric fruits

The classification of fleshy fruits as either climacteric or non-climacteric is based on the hormonal mechanisms that control the ripening process and respiratory response (Bouzayen et al., 2010; Forlani et al., 2019). The characteristic increase in respiration and rapid rise in ethylene production observed in climacteric fruits at the onset of ripening serves as a central signal for initiating and coordinating the ripening process. In contrast, non-climacteric fruits do not exhibit an increase in respiration and ethylene plays a minor role in the ripening process, although there are some ethylene-dependent processes in this type of fruit (Adams-Phillips et al., 2004; Bouzayen et al., 2010). Additionally, non-climacteric fruits have a lower number of ethylene receptor genes compared to climacteric fruits (Chen et al., 2018). In contrast, abscisic acid is regarded as the principal hormone governing the ripening process in non-climacteric fruits (Chen et al., 2020; Jia et al., 2020; Perotti et al., 2023). Additionally, other plant hormones, including IAA, gibberellic acid, cytokinin, methyl jasmonate, and brassinosteroids, have been shown to influence specific aspects of fruit ripening (Brumos, 2021).

The distinction between climacteric and non-climacteric fruits extends beyond the hormonal regulation of fruit ripening; it also encompasses the molecules accumulated during fruit growth. The accumulation of elevated levels of starch during fruit development is a distinctive trait of climacteric fruits (Yu et al., 2022), whereas non-climacteric fruits exhibit only transient storage and accumulate relatively minimal amounts of starch.

In the case of the banana, the fruit accumulates considerable amounts of starch in the pulp (20%–25%) during fruit development (Xiao et al., 2018). This strategy of climacteric species has the advantage that starch biosynthesis is likely to increase the sink strength of the fruit. Bananas are typically harvested while still immature, and the starch is degraded during postharvest ripening. This process provides the requisite carbon and energy reserves for the production of sucrose and other quality-related metabolites (Xiao et al., 2018; Yu et al., 2022). Consequently, bananas undergo a period of rapid fruit growth with starch accumulation, followed by a catabolic process that releases sugars when the fruit stops growing. This strategy effectively minimizes osmotic perturbations in the cells (Dong and Beckles, 2019). In contrast, non-climacteric fruits, such as grapes and loquats, have been observed to exhibit both elevated sugar accumulation and accelerated fruit growth at the onset of ripening (Gariglio et al., 2003a; Matthews et al., 2009).

In non-climacteric fruits, this scenario intensifies the water relations between internal and external tissues in developing fruits, thereby imposing additional stress on growth. In a similar context, Zhang and Keller (2016) demonstrated that the accumulation of sugar in ripening grape berries requires the removal of excess phloem-derived water, either by transpiration or xylem backflow, in order to prevent the occurrence of cracks. For example, the restriction of both xylem backflow and transpiration resulted in a twofold increase in the incidence of cracks in Concord and Syrah grapes (Zhang and Keller, 2016). It may therefore be hypothesized that xylem backflow plays a more significant role in non-climacteric fruits in order to compensate for the absence of starch reserves, thereby increasing fruit sink strength. Furthermore, it may be postulated that xylem backflow replaces the absence of starch reserves in non-climacteric fruits as a vital energy source for the synthesis of quality-related metabolites during ripening, while reducing the risk of osmotic disturbance. However, these hypotheses require further investigation.

### Environmental factors

The primary environmental factor contributing to fruit cracking is water, with both soil moisture and water on the fruit surface playing a significant role. The incidence of cracking is heightened by rainfall that affects crops that were previously subjected to water stress (see the *Fruit growth rate* section). Furthermore, inadequate irrigation practices during fruit ripening increase the probability of fruit cracking (Mesejo et al., 2016; Wang et al., 2021).

In tomato, the results of Ikeda et al. (1999) suggest that the water potential gradient between the flesh and the water source is more closely related to the incidence of fruit cracking than the water relations between the flesh and the skin tissues, but it is noteworthy that although the treatments in their experiment significantly altered the growth rate of the fruit, the effect on cracking was not thoroughly discussed.

In the case of loquat, prolonged deficit irrigation before harvest has been demonstrated to reduce the incidence of purple spot (Jiménez et al., 2022). However, it should be noted that temperature and radiation represent the primary environmental factors influencing the incidence of purple spot (Gariglio et al., 2003b).

The effect of light exposure on fruit is significant in terms of the incidence of cracking and purple spot. It has been demonstrated that fruits exposed to sunlight, particularly on the sun-facing side, are more susceptible to cracking (Ulinnuha et al., 2020) and purple spot (Gariglio et al., 2003b). However, the precise impact of light on the incidence of cracking remains unclear (Fischer et al., 2021). In the Mediterranean basin, it has been observed that solar radiation increases loquat fruit temperature by up to 8°C, thereby increasing fruit sink activity. Consequently, this increased sink activity results in increased flesh sugar availability and an accelerated fruit growth rate, thereby rendering the fruit more susceptible to purple spot (Gariglio et al., 2008b). Similarly, low night temperatures have been observed to contribute to an increased incidence of purple spot. When loquat is grown in a greenhouse with controlled minimum night temperatures above 15°C, an increase in the concentration of sugars in the skin has been observed. This phenomenon can be attributed to increased fruit activity during the night, which favors the partitioning of assimilates into the skin and reduces the risk of purple spot (Gariglio et al., 2008b).

### Other factors

A close relationship was found between fruit splitting and soil texture in citrus. Results showed an inverse correlation between clay and silt soil content and fruit splitting, while sand content was positively correlated with a reduced incidence of splitting. It was also found that the incidence of splitting was found to be higher in trees with larger xylem vessels in the peduncle, which was attributed to the rootstock. The “Carrizo” and “C-35” citrange rootstocks exhibited a higher incidence of splitting than the “FA-5”, “Cleopatra”, and *Poncirus trifoliata* rootstocks. Furthermore, reducing irrigation frequency by half resulted in a 5°C increase in canopy midday temperature and a 15% increase in splitting. The authors conclude that irregularities in the tree water status, due to interactions between soil moisture, rootstock, and climatic conditions, result in significant changes in fruit growth rate and increased incidence of fruit splitting (Mesejo et al., 2016).

The propensity for fruit to crack is genetically influenced, with different cultivars exhibiting varying degrees of susceptibility. This process is regulated by a combination of genes, rather than a single gene (Wang et al., 2021). For example, the *SlGH9-15* gene, which plays an important regulatory role in cellulose activity, has been identified as a major factor associated with fruit cracking in tomatoes. Therefore, tomatoes prone to cracking had increased cellulase activity and decreased cellulose content, especially at the red ripening stage (Lin et al., 2023). Similarly, the prevalence of purple spot is significantly influenced by cultivar, with early maturing cultivars showing increased susceptibility (Shah et al., 2023).

The occurrence of fruit cracking is linked to changes in the biochemical properties of the cuticle, epidermis, and hypodermis. Recent research has identified genes involved in cell wall modification, cuticular wax biosynthesis and transport, cuticular membrane deposition, and associated transcription factors, providing invaluable insights into the genetic basis of this phenomenon (Santos et al., 2023). However, despite this progress, there remains a need to integrate these genetic findings into a unified hypothesis that elucidates the regulatory mechanisms governing fruit cracking.

## Concluding remarks

This review puts forth a hypothesis regarding the alterations in water relations between internal and external tissues in developing fruit, situated within the context of the epidermal growth control hypothesis. The pressure potential of the outer tissues is a consequence of the expansion of the inner tissues, rather than osmotic water uptake, as observed in the flesh. This results in a false turgor, which, when considered with the osmotic potential of the skin, can be used as an indicator of the level of growth stress and the risk of skin cell dehydration. The primary factors identified as potential determinants of the water balance between internal and external tissues include the growth rate of the fruit, the gradient of sugar concentration (on a dry weight basis) between the flesh and skin tissues, the proportion of apoplastic and symplastic solutes, and the strength of the skin. These factors render the fruit susceptible to physiological disorders associated with water relations. While the relevance of skin water potential and its components to the hypothesis presented may be validated in future research, it is recommended that they be included in studies of physiological disorders under different predisposing conditions to improve our understanding of the physiological process involved.
